# Green and low-cost synthesis of zinc oxide nanoparticles and their application in transistor-based carbon monoxide sensing

**DOI:** 10.1039/d0ra00478b

**Published:** 2020-04-02

**Authors:** Ashwath Narayana, Sachin A. Bhat, Almas Fathima, S. V. Lokesh, Sandeep G. Surya, C. V. Yelamaggad

**Affiliations:** Department of Bio-Medical Engineering, Rajiv Gandhi Institute of Technology Cholanagar, R. T Nagar, Hebbal Bengaluru India; Centre for Nano and Soft Matter Sciences P. B. No. 1329, Prof. U. R. Rao Road, Jalahalli Bengaluru India yelamaggad@gmail.com; Department of Nanotechnology, Centre for PG Studies- Bangalore Region, Visvesvaraya Technological University Muddenahalli Chikkaballapur India lokeshsampangi@gmail.com; Electrical Engineering Department, Indian Institute of Technology Bombay Mumbai India

## Abstract

There has been steady progress in developing reliable and cost-effective strategies for the clean production of zinc oxide (ZnO) nanoparticles (NPs) owing to their unique structural and wide functional characteristics. While the green synthesis of such NPs from plant extracts has emerged as a sustainable and eco-friendly protocol, it is greatly restricted owing to the scarcity of potential natural precursors necessitating comprehensive investigations in this direction. Herein, we report a facile, low-cost green synthesis and characterization of ZnO NPs along with the demonstration of their usage as an active media in organic field-effect transistor (OFET) devices for sensing carbon monoxide (CO) gas. The ZnO NPs obtained from *Nelumbo nucifera* (lotus) leaf extract-mediated solution combustion synthesis at a much lower initiation temperature, the first of its kind, were characterized by various techniques such as UV-vis spectroscopy, XRD, EDX analysis, TEM and FESEM. The data derived from these experiments clearly evidence the formation of very pure and crystalline ZnO NPs possessing nearly spherical-shape with a size of 3–4 nm. The p-type organic field-effect transistor (OFET) device, fabricated using poly(3-hexylthiophene-2,5-diyl) (P3HT) and ZnO NPs, showed a field-effect mobility of 10^−2^ cm^2^ V^−1^ sec^−1^ with a slightly enhanced response of detecting CO gas at room temperature (RT). The phenomenon was further confirmed by the variation in electrical parameters of the OFET such as field-effect mobility (*μ*), on-current (*I*_on_), and off-current (*I*_off_). The selectivity and sensitivity of the fabricated device in CO gas detection was found to be more prominent than the other reducing gases (hydrogen sulphide, H_2_S and ammonia, NH_3_) and methanol vapours tested.

## Abstract 2

An OFET-based CO gas sensor has been demonstrated where ZnO NPs realized by an inexpensive, environmentally friendly method have been employed as an active medium.

## Graphical abstract

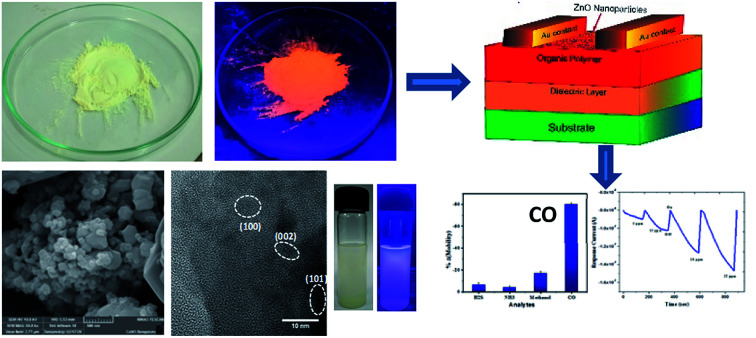

## 1. Introduction

Nanomaterials display a wide range of unique physicochemical properties that are well-known to originate from the high surface area and nanoscale size of their constitutional components, called nanoparticles (NPs).^1–6^ In fact, owing to their intrinsic and genuine material characteristics, they have been regarded as ideal substances in diverse areas of applied science which include catalysis,^1–3^ electronics,^4–6^ photonics,^7–9^ imaging,^10^ field emission displays,^11,12^ energy harvesting,^13,14^ energy-storage,^15–17^ cosmetics,^18,19^ drugs and medication,^20^ agriculture,^21,22^ environment,^23^ and related disciplines. However, at present, there has been a tremendous upsurge of interest in the synthesis and characterization of novel NPs capable of exhibiting semiconductor properties.^24–26^ Such interest originates from the fact that the varied properties of semiconductor nanomaterials hold huge promise for many emerging technological regimes such as solar cells,^27^ nanophotonics,^7–9^ nanoelectronics,^4–6^ catalysis,^1–3^ light-emitting diodes,^28,29^ laser technology,^30,31^ chemical,^32,33^ biosensing,^34,35^ energy-conversion,^36,37^ miniaturized devices,^38,39^*etc.*

Among a large variety of semiconductor nanomaterials known, zinc oxide (ZnO) NPs have seemingly secured a special place in nanomaterials research and technological domains. This is not surprising given the fact that ZnO possesses, at room temperature, a direct wide bandgap (3.37 eV), large exciton binding energy (60 meV) and large bond strength.^40–42^ As an archetypal n-type semiconductor material, ZnO has been specially considered as the promising resistive-type active media for sensing a plethora of gaseous and bio systems.^43^ The occurrence in a wide range of morphologies (shapes), such as wires, needles, urchins, nanorods, sphere, ellipsoids, helices, combs, flowers, *etc.*,^44^ enables gaining control over the surface to volume ratio and thus, enhances the utility of ZnO nanomaterials in different sensing applications. Furthermore, ZnO has certain remarkable features such as chemical/thermal stability,^45,46^ sensitivity and selectivity.^47,48^ Besides, when compared to other metal oxide NPs, ZnO nanomaterials are not only inexpensive but also relatively non-toxic (harmless/safe) and environmentally benign.^49,50^ Therefore, currently, there has been significant interest in the clean and cost-effective synthesis of ZnO NPs, and exploration of their uses as an active media in many device applications.

Over the years, a wide number of physical, chemical and hybrid synthetic strategies have been developed and employed to obtain ZnO NPs.^50–59^ However, these approaches appear to suffer through certain serious setbacks, such as the usage/emission of highly toxic and hazardous materials, impurities (leading to biological risking), inconsistent yields/size/morphology, high temperature/pressure conditions, *etc.* On the other hand, green synthetic procedures, using either plant (extract) or biological species (microorganisms, & enzymes), are gaining importance as they are simple (generally single-step processes), produce clean nanomaterials, inexpensive, safe and eco-friendly. In fact, these methods, when compared to the other protocols, yield ZnO NPs with well-defined size and morphology.^60–67^ Although the methods of obtaining ZnO NPs from plant extracts appear to have notable advantages over the other ones, the true potentials of the latter sources have not been fully explored and revealed due to the lack of systematic research approach necessitating comprehensive research work in this direction. In light of these observations, for some time now, we have been working in this interesting domain.^6,68^ In continuation of our studies in this direction we herein report, a simple, cost-effective and environmentally sustainable green approach for the synthesis of ZnO NPs using an aqueous extract of *Nelumbo nucifera* (lotus) leaves. The ZnO NPs thus obtained at lower initiation temperature (∼150 °C for 30 min), the first of its kind, were characterized with the aid of ultraviolet-visible (UV-vis) spectroscopy, X-ray diffraction (XRD), energy dispersive X-ray (EDX) analysis, electron microscopy (TEM) and field emission scanning electron microscopy (FESEM). The ZnO NPs along with P3HT were used in fabricating the OFET device that senses CO gas with notable sensitivity and selectivity. The aforesaid aspects pertaining to the synthesis, characterization and transistor performance are presented below.

## 2. Materials and methods

### 2.1. Chemicals

The requisite AR grade solvents such as ethanol and isopropyl alcohol (IPA) were purchased from Merck. Poly(3-hexylthiophene-2,5-diyl) (P3HT) was procured from Sigma Aldrich, whereas zinc nitrate, Zn(NO_3_)_2_·6H_2_O was purchased from Fisher Scientific.

### 2.2. Preparation of aqueous lotus leaf extract

Freshly plucked *Nelumbo nucifera* leaves were placed under running water for a while and then gently wiped with a piece of new (clean) white banian cloth. After blowing air gently with aid of an air-gun at RT, the lotus leaves were torn to small pieces by hands protected with surgical clean gloves. About 25 grams of the leaf pieces were immersed in 100 ml of deionized (DI) water contained in a clean, 200 ml beaker. The heterogeneous mixture was placed on top of a hot plate-magnetic stirrer and heated at 75 °C with constant stirring for 3 h where the beaker was partially closed with a watch glass. The extract thus obtained was filtered repeatedly using Whatman filter paper grade 1 (particle retention ∼11 μm) and cooled, affording a deep dark-brown colored solution of lotus leaf extract. This was used for the synthesis of ZnO NPs as described below.

### 2.3. Synthesis of ZnO NPs

**Fig. 1:**
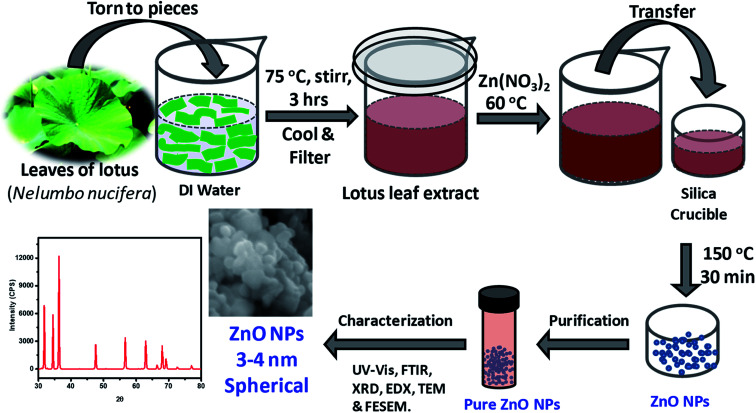
Schematic illustration of the steps followed in the green synthesis of ZnO NPs from aqueous extracts of *Nelumbo nucifera* leaves, and their characterization.

As depicted in Fig. 1, ZnO NPs were prepared by a green synthesis approach *via* a solution combustion method using lotus leaf extract. Initially, 0.1 M aqueous solution was prepared by adding 0.595 g of zinc nitrate, Zn(NO_3_)_2_·6H_2_O in 20 ml of DI water under constant stirring at RT. The resultant aqueous Zn(NO_3_)_2_ solution was added drop-wise to the 20 ml of leaf extract at 60 °C. It is worth mentioning here that the usage of aforesaid quantities/concentration consistently yielded ZnO NPs quantitatively without much residual impurities. The resultant mixture was transferred to silica crucible and heated at 150 °C using an electric Bunsen burner for ∼30 min which lead to the formation of ZnO NPs where Zn(ii) ions react with oxygen present in the media and leaving the other components to evaporate/burn. The crude ZnO NPs thus formed were purified further by treating them with DI water and ethanol as described below. The crude product obtained was poured into 20 ml of DI water and then, resultant mixture (suspension) was centrifuged, the residual supernatant was discarded; this process was repeated for 4 times additionally. The product obtained was then poured into 20 ml of rectified spirit (ethanol) and sonicated for a while; the supernatant was discarded; this process was repeated 5 times additionally. Pure ZnO NPs thus obtained were thoroughly dried and stored in an amber sample glass bottle at RT.

## 3. Results and discussion

### 3.1. Structural characterization

**Fig. 2:**
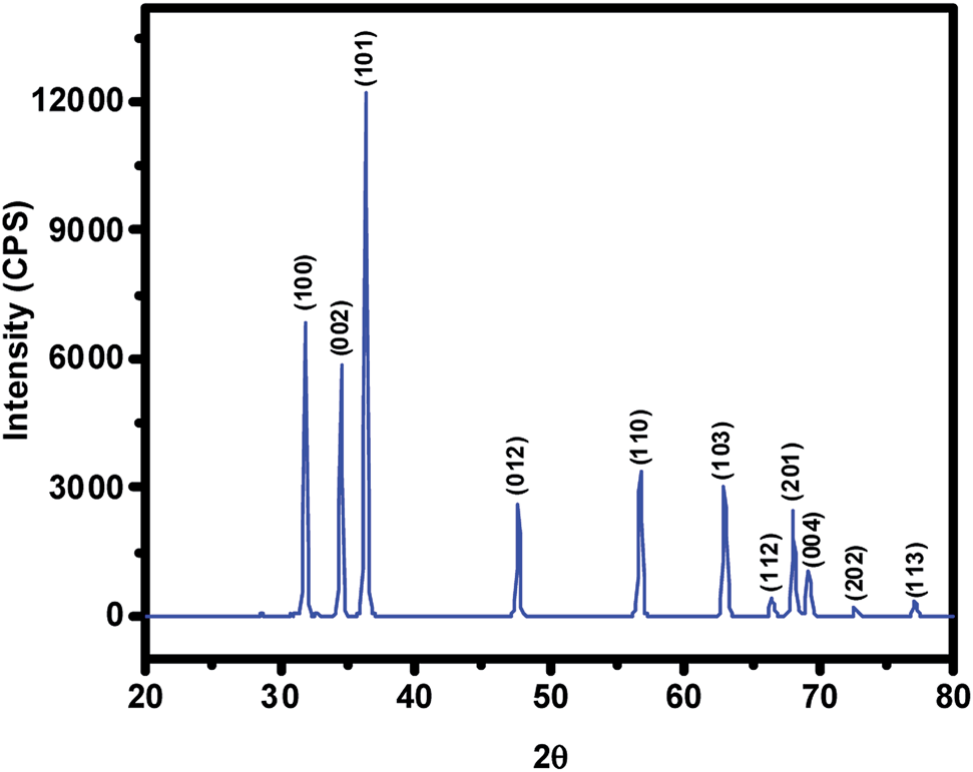
The intensity *vs.* 2*θ* profile obtained for the ZnO NPs prepared from aqueous extract of *Nelumbo nucifera* leaves. Note that this pattern clearly suggests the hexagonal crystal structure of the synthesized ZnO NPs belonging to *P*6_3_*mc* space group.

The ZnO NPs obtained in this study from the green synthetic approach were fully characterized with the aid of several spectroscopic and microscopic methods. To begin with, the powder XRD technique was employed not only to ascertain the crystalline structure but also to figure out the crystallite size of particles from the width (broadening) of the X-ray peaks. XRD pattern of the as-synthesized ZnO NPs recorded at room temperature, in the 2*θ* range of 20–80° using Rigaku Ultima IV X-ray diffractometer, has been shown in Fig. 2 where several well-resolved diffraction peaks can be seen. The prominent peaks occurring along (100), (002), (101), (012), (110), (103), (112), (201), (004), (202) and (113) crystallographic planes correspond respectively to the Bragg reflections at 2*θ* values of 31.8°, 34.5°, 36.3°, 47.6°, 56.7°, 62.9°, 66.0°, 68.0° 69.2°, 72.7° and 77.0°. These data along with spacing (*d*) vales have been listed out in Table 1; in fact, XRD data obtained was found to be in good agreement with that of the reported ones for the ZnO NPs. The absence of any other uncharacteristic/extra peak(s) clearly vouches for the purity of ZnO NPs accomplished through biosynthesis. In particular, the presence of intense peaks for the (100), (002) and (101) planes indicates highly crystalline and single-phase ZnO NPs. The indexing powder XRD data implied that sample has the zincite crystal structure of space group *P*6_3_*mc* belonging to hexagonal crystal lattice system (DB card number: 1011258) where the known correlations between the length of the unit cell axes and the angles respectively are *a* = *b* ≠ *c*, *α* = *β* = 90°, *γ* = 120°. The measured cell parameters obtained from the data were found to be *a* = *b* = 3.26 Å and *c* = 5.22 Å. The crystallite size (*D*) of the ZnO NPs was figured out from the broadening of diffraction peak *viz*, 001 and 100 with the aid of Scherrer's formula: *D* = *kλ*/(*β* cos *θ*) where *λ* is the wavelength of light used (CuKα radiation, 1.54 Å), *k* is the Scherrer's constant (0.94), *β* is the full width at half maximum of the selected diffraction peaks corresponding to planes, *θ* is the Bragg's angle obtained from 2*θ* value; *D* was found to be 26 nm.

**Table 1:** The data derived from XRD pattern recorded at RT for the pristine ZnO NPs obtained from the aqueous extract of *Nelumbo nucifera* leaves

S. no	Peak positions (2*θ*) in degree	Crystallographic planes (*hkl*)	*d* (Å)
01	31.8	(100)	2.80
02	34.5	(002)	2.59
03	36.3	(101)	2.46
04	47.6	(012)	1.90
05	56.7	(110)	1.62
06	62.9	(103)	1.47
07	66.0	(112)	1.40
08	68.0	(201)	1.37
09	69.2	(004)	1.29
10	72.7	(202)	1.23
11	77.0	(113)	1.18

**Fig. 3:**
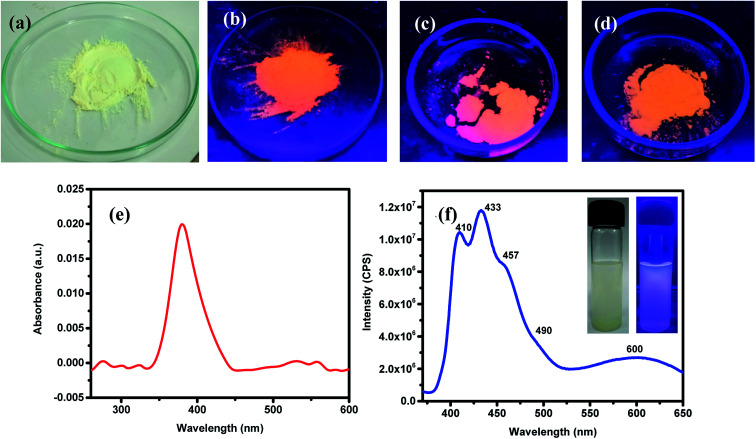
Digital photographs of the green-synthesized ZnO NPs seen under normal light (a). Fluorescence images of the ZnO NPs seen when exposed to UV-lamp of 365 nm: (b) as-synthesised (at 150 °C), (c) annealed at 400 °C and (d) annealed at 600 °C. Optical absorption (UV-vis) (e) and emission (f) spectra of the as synthesized ZnO NPs dispersed in ethanol; here, insets shows digital photographs of the ZnO NPs, dispersed in EtOH, illuminated with ordinary (left panel) and UV-lamp of 365 nm (right panel); in the latter, blue luminescence is apparent.

ZnO NPs are well-known to exhibit some remarkable photophysical properties. Thus, the ZnO NPs realized in this study were investigated for their absorption and emissive characteristics. Fig. 3a and b respectively show the digital photographs of the synthesized (pristine) NPs captured under normal light and UV-light of 365 nm. It is quite interesting to note that ZnO NPs appear orange when exposed to UV light. The photophysical behavior of sonicated suspension of ZnO NPs in ethanol was investigated by recording the optical absorption and emission spectra. Fig. 3c depicts the room temperature UV-vis absorption spectrum recorded in the wavelength region of 200–600 nm. The distinct absorption maxima existing around 380 nm corresponds to ZnO NPs which arises due to large excitation binding energy; this is in good agreement with the reported results that ZnO NPs show optical absorption in the range of 360–380 nm. This peak with maxima at 380 nm can be assigned to the surface plasmon resonance (SPR) of ZnO NPs wherein the incident light is absorbed for the collective oscillation of free conduction band electrons.^69,70^ The photoluminescence spectrum recorded for an excitation wavelength of 350 nm has been presented in Fig. 3b where two strong peaks at 410 and 433 nm with a shoulder at 457 nm and a broad peak at 600 nm can be seen. Thus, all emission peaks of ZnO NPs emerging in the visible (400–600 nm) region have been ascribed to different intrinsic defects.^69,70^ The insets of Fig. 3b are the digital photographs of ZnO NPs (dispersed in EtOH) seen under an ordinary lamp (left) and UV (365 nm) lamp where the blue fluorescence can be seen (right).

**Fig. 4:**
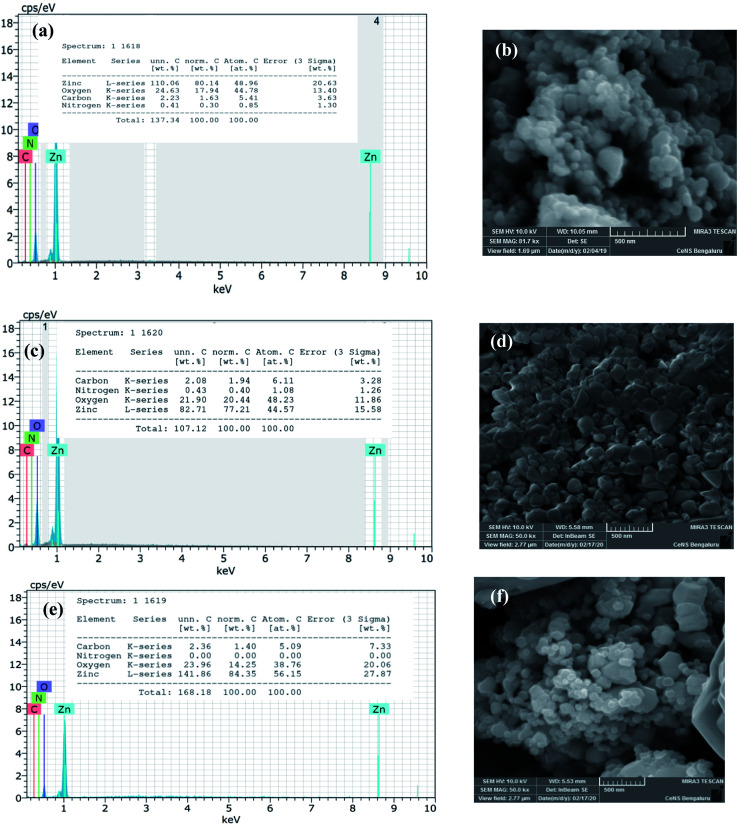
The data derived from EDAX-FESEM studies of green synthesized ZnO NPs: EDAX profile: (a) as-synthesized, (c) annealed at 400 °C and (e) annealed at 600 °C & (b) FESEM images of as synthesized ZnO NPs; (d) annealed at 400 °C and (f) annealed at 600 °C.

The biosynthesized ZnO NPs were subjected to EDAX and FESEM studies to ascertain the atomic percentage of the elemental composition and surface morphology/size/shape respectively. The freshly sonicated ethanolic solution of NPs was drop cast on silicon (Si) surface and air-dried and used for FESEM and EDAX analysis. Fig. 4a illustrates the EDAX spectrum where the elemental composition of the ZnO NPs can be found. This spectrum not only confirmed the presence of the Zn and O elements but also evidenced the relative purity of the material synthesized. Precisely, the EDAX analyses showed the elemental composition of Zn with 80.1% and O with 17.9%; however, the theoretical stoichiometric mass% of Zn and O are 80.3% and 19.7% respectively. Needless to say, the deviations in the experimental data seen from that of the theoretical one can be attributed to the presence of some organic residue and/or other elements coming from the substrate in minute quantities. The topographic image recorded is shown in Fig. 4b where the presence of thickly aggregated spherical ZnO NPs spread over many regions can be observed. The size distribution histogram revealed that green synthesized ZnO NPs are in the range of 20–40 nm. In order to reveal the effect of thermal annealing on the NP size, the synthesized ZnO NPs (at 150 °C) were subjected to heating at elevated temperatures. The pristine sample was especially annealed at two chosen temperatures *viz.*, at 400 °C and 600 °C. The annealed ZnO NPs were characterized with the help of FESEM-EDAX technique. Fig. 4c and e depict the EDAX spectra of the annealed ZnO NPs. As can be seen from the FESEM images, shown in Fig. 4d and f, the agglomerated NPs separate out upon annealing. These images also reveal that the individual NPs are of varying sizes ranging between 20–50 nm. The comparison of the EDAX spectral results of the annealed samples with that of the as synthesized NPs clearly indicates the oxygen content in the annealed NPs varies to some extent. However, the carbon content remains unaltered meaning that the organic residue remains in similar traces in all the samples.

**Fig. 5:**
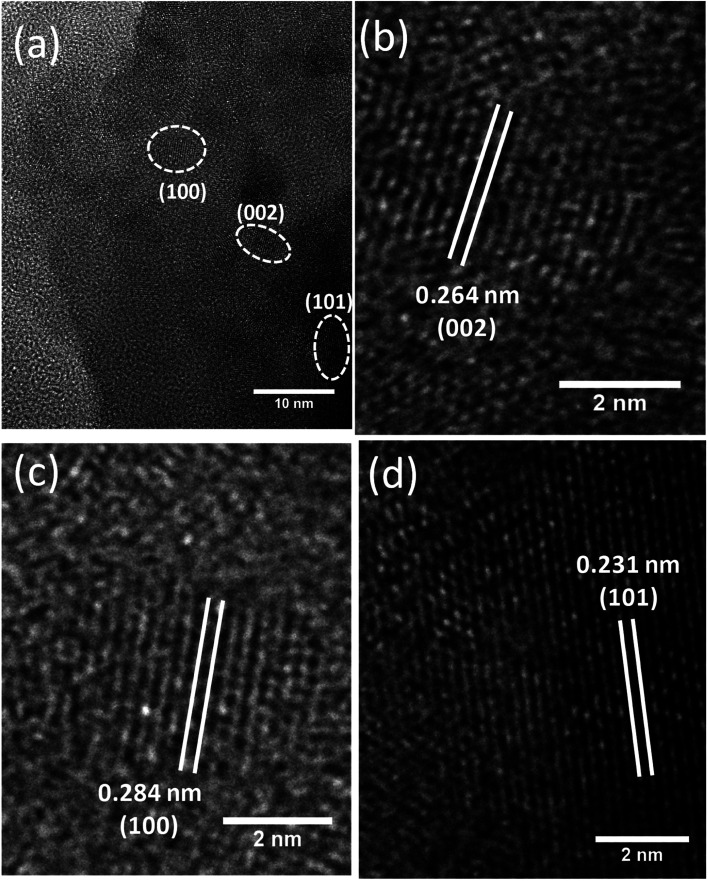
(a) HRTEM images of green synthesized ZnO NPs. (b–d) The magnified images of certain chosen portions of the image (a), which were utilized to figure out the spacings (*d*) of (100), (002) and (101) crystallographic planes.

The HRTEM analysis was performed not only to corroborate the abovementioned findings from XRD & FESEM techniques but also to known the nanostructural feature of the ZnO NPs realized from the green approach. For this experiment, ZnO NPs were dispersed in ethanol by sonication, and a droplet of which was placed onto a 400-mesh carbon-coated copper grid and dried. An image obtained from this sample has been shown in Fig. 5a. It is immediately apparent from the image that lattice fringes of varying orientations exist. This image also hints at the agglomeration/aggregation of NPs that can be attributed to the lack of sufficient energy needed for the SPR of lattice planes of ZnO particles. Using the image shown in Fig. 5b, the spacing (*d*) between two consecutive lattice planes was calculated, and it was found to be 2.8 Å which agrees with the one calculated from XRD. Likewise, the *d* values of (002) and (101) planes calculated, from the images shown in Fig. 5c and d, were found to be 2.64 Å and 2.31 Å respectively; of course, these values differ slightly from the ones obtained from XRD.

### 3.2. Device fabrication and characterization

#### 3.2.1. Fabrication of OFET

**Fig. 6:**
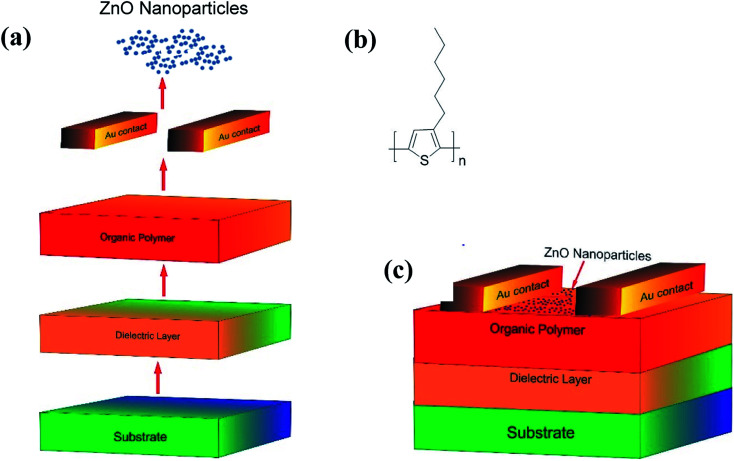
(a) Steps involved while making OFET device; (b) the molecular structure of P3HT; (c) device structure of the fabricated OFET sensor.

Schematic representation of the steps involved, the molecular structure of P3HT used in the fabrication and a p-type organic field-effect transistor (OFET) fabricated have been shown in Fig. 6a, b and c respectively. Initially, a single side polished n-type (100) Si wafer with a resistivity (0.01–0.02 Ω cm) was coated with SiO_2_ dielectric layer of 300 nm thick. The Si wafer, after cutting into the desired dimension (2 × 2 cm), was cleaned repeatedly with DI water followed by acetone and isopropyl alcohol (IPA). To ensure that no liquid (water/solvent) traces are adhering, the specimen was heated at 120 °C for ∼5 minutes. In the mean-time, P3HT (Fig. 6b) was dissolved in 1,2-dichlorobenzene to make a solution of 2 mg ml^−1^; this solution, after subjecting to sonication for a while, was heated at 70 °C for ∼45 minutes and cooled to get a homogeneous solution. The Si wafer was purged with nitrogen upon placing it on the spin coater stage to remove traces of dust. The Si wafer was placed on the spin-coater, cleaned with acetone and IPA using lint-free swabs, and then coated with the drop cast P3HT solution. The plate was spun in two stages, first at 500 rpm for 5–10 seconds and then at 1500 rpm for 60 seconds. This spin-coating technique ensured the uniform distribution of the sample on the substrates with a coating thickness of ∼100 nm. After annealing at 90 °C for 60 min, the gold (∼60 nm) was then sputtered on top of the P3HT layer using a physical mask at 10^−6^ mBar and as discussed in our previous work.^71^ The resultant substrate was further spin-coated with a suspension of ZnO NPs (2 mg dispersed in 1 ml of EtOH) at 1500 rpm for ∼45 seconds; it may be mentioned here that the ZnO coating serves as the receptor layer. The schematic diagram of the OFET device eventually made has been shown in Fig. 6c.

#### 3.2.2. Characterization

**Fig. 7:**
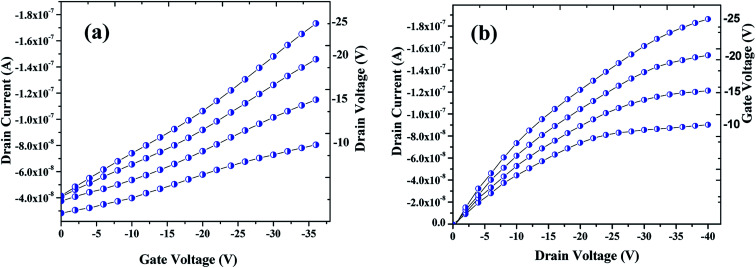
Profiles showing the transfer (a) & drain (b) characteristics of the OFET device fabricated that are archetypal electrical characteristics of a p-channel.

The electrical performances of the OFET devices fabricated were examined at a probe station by using source meter. The transfer and output (drain) characteristics of the OFET device are shown in Fig. 7a and b respectively. Seemingly, these plots are typical of a p-type transistor behavior of an OFET device. As can be seen in Fig. 7a, drain current (ID) was measured by varying the gate voltage (*V*_GS_) from 0 V to −36 V with an interval of −2 V, while the drain voltage (*V*_DS_) was varied from −10 V to −25 V with step of −5 V. Mobility (*μ*) was also calculated from the plot that was found to be in the range of 10^−2^ cm^2^ V^−1^ sec^−1^. As shown in Fig. 7b, the drain current were plotted by varying the drain voltage from 0 V to −40 V with step of −2 V and varying the gate voltage from −10 V to −25 V with step of −5 V. Fig. 7b clearly shows a linear increase of the drain current with drain voltage at linear region which then saturates with the higher drain voltage owing to the pinch-off of the accumulation layer, the drain current saturation (IDS) was found approximately over ∼1.87 × 10^−7^ A. Devices were tested in an ambience humidity of 60% RH for both exposed and unexposed gases.

**Fig. 8:**
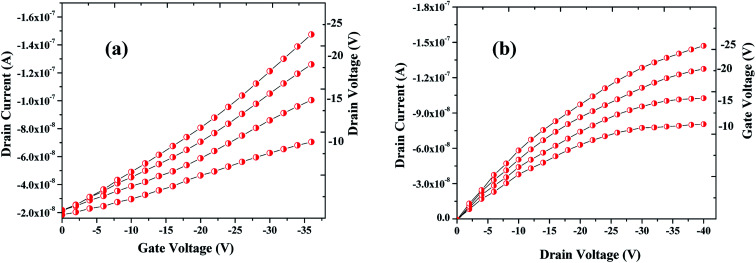
Profile showing the transfer (a) & drain (b) characteristics of the OFET device after exposing it to the CO gas. Notice that although the overall characteristics of the device is similar to the one seen before the exposure to CO gas (see Fig. 7a and b) but notable changes in the ON-current and mobility are observed when the device was exposed to CO analyte.

**Fig. 9:**
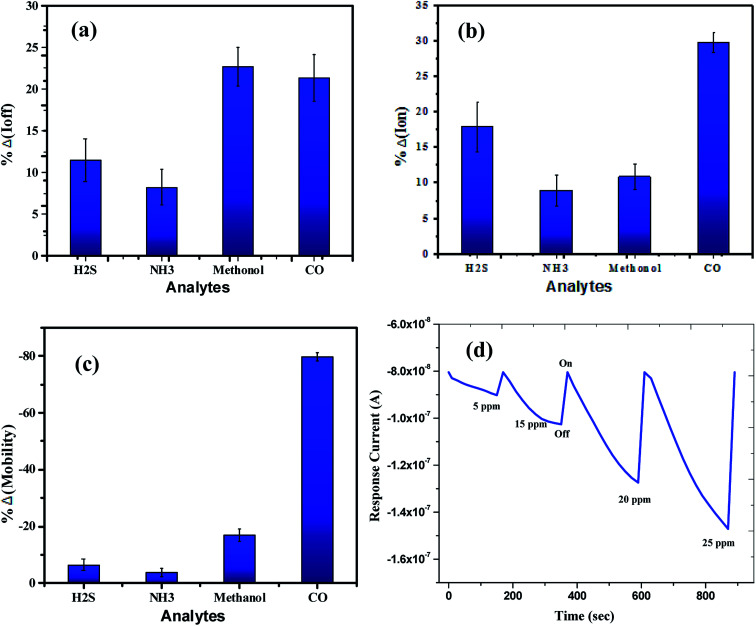
The overall sensor response for the analytes (H_2_S, NH_3_, & CO gases) and methanol vapours tested: (a) off current response, (b) on current response and (c) mobility. (d) The relationship of drain-source current as a function of time for different concentrations of the CO analyte at *V*_GS_ = −25 V and *V*_DS_ = −40 V.

The sensing tests were carried out on these fabricated OFETs by exposing them to certain selected gases and the vapors of methanol (CH_3_OH). In particular, gases such as carbon monoxide (CO), hydrogen sulphide (H_2_S) and ammonia (NH_3_) were used for the sensing studies. The concentrations (exposure limits) of gasses were maintained as per occupational safety and health administration (OSHA) standards and measurements were carried out at RT. The 100 ppm of CO cylinder with 2 MFCs were considered to perform sensing test by exposing the device to CO gas of 25 ppm concentration, and the responses thus obtained have been shown in Fig. 8a and b. While the overall characteristics of the devices before (Fig. 7a) and after exposure to CO gas (Fig. 8a) remains identical, a significant change in the ON-current was observed only upon exposing the device to CO gas. The calculations carried out from the profiles shown in Fig. 7a and 8a evidence the expected changes in the mobility values. The accumulation of abundant positive charges, which is evident from Fig. 8b, suggests an excellent performance and consistency of the P3HT channel layer. The substantial change in the electrical conductivity of the device evidently implies the effective/strong interaction among the surface (ZnO NPs layer) and CO molecules. Besides, the drop in transfer and output characteristics of the device exposed to the gas (Fig. 8a and b), when compared to that of unexposed one (Fig. 7a and b), is immediately noticeable and convincing. These results clearly reveal the ability and slightly enhanced response of the fabricated device in sensing CO gas at RT where the ZnO NPs serve as the receptor layer. To test the analyte-selectivity of the fabricated device for sensing, similar experiments were carried out on hazardous H_2_S and NH_3_ gases as well as on the toxic methanol vapours. The results obtained from these experiments were compared with those obtained for CO gas. Fig. 9a, b and c depict the changes respectively occurring in the off-currents, on-currents and mobility of analytes H_2_S and NH_3_ gases and methanol vapours tested with the same concentrations that of CO gas considered. As can be seen in Fig. 9a and b, the percentile OFF and ON currents are higher for the CO gas as compared with the other reducing gases; that is the slightly enhanced response is promising in the case of CO gas. Similarly, as can be found in Fig. 9c, the change in mobility is much higher for CO gas as compared with other gases. Fig. 9d shows the variation in drain-source current as a function of time for different concentrations *viz.*, 5, 15, 20 and 25 ppm of the CO analyte. The transient analysis of the OFET sensor was recorded at *V*_GS_ = −25 V and *V*_DS_ = −40 V, which shows no decrease in drain-source current with time. During this response process, the OFET device was introduced with CO concentration for approximately 3 min, and recovery was initiated by introducing nitrogen until the drain-source current recovers to the actual value. When CO was introduced, the reduction in the drain-source current of OFET sensor was clear indication of CO detection. However, the complete recovery of the drain-source current was done after the device was again purged with nitrogen. Furthermore, the CO concentration was varied as 5, 15, 20 and 25 ppm for which the drain-source current was significantly found decreasing with increasing CO concentration and there was significant increase in recovery time as well. We calculated the sensitivity as per the calculations in our previous papers.^6^

#### 3.2.3. CO sensing mechanism

A literature survey indicates that the CO gas has a higher adsorption tendency towards ZnO (bulk/NPs) surface as compared with other competing gasses, which makes ZnO a potent sensor for CO detection.^72–74^ Therefore, in the present study, the OFET devices have been fabricated using the green-synthesized ZnO NPs, especially for the sensing CO gas. Unlike many other gaseous interactions with the surface, CO interaction onto the ZnO surface cannot be explained solely by single charge transfer reaction.^71^ It is observed that the surface of ZnO gets partially reduced when exposed to CO molecules. In particular, the lattice oxygen on the surface of ZnO gets reduced with CO and forms chemisorbed species *i.e.*, CO_2_ (adsorbed) δ^−^ and also forms positively charged oxygen vacancies.^72–74^ These oxygen defects act as a donor and its degree of ionization depends on the Fermi level.^73^ The accumulation of positively charged oxygen defects,^38^ as well as the chemisorbed CO_2_, which is partially negatively charged, will bring about a substantial difference/variation in the surface potential of the device. In other words, these variations on the surface of ZnO will bring about remarkable changes in the electronic properties. That is, the variation in the electron density in the ZnO layer, specifically formation of defect states upon interaction with CO will bring about the change in hole concentration and mobility of the P3HT layer. The recombination of the electron–hole pair among these two layers will lead to a decrease in the drain current of the device. In essence, upon exposure to CO gas, significant changes are observed in the OFET device characteristics due to CO adsorption and partial reduction of ZnO. This study demonstrates that ZnO (as an active receptor media)-P3HT (channel layer) field-effect transistors can be used effectively for the detection of CO molecules selectively.

## 4. Conclusion

An OFET-based CO gas sensor has been fabricated using ZnO NPs synthesized by an inexpensive environmentally friendly method. Specifically, an aqueous extract of *Nelumbo nucifera* (lotus) leaves has been treated, for the first time, with Zn(NO_3_)_2_ to obtain ZnO NPs in almost pure form. One of the attractive features of this green approach, which involves solution combustion synthesis is that the initiation temperature is much lower (∼150 °C) when compared to ones reported in the literature. The determinations of chemical composition, purity, surface morphology, shape, size, photophysical properties *etc.*, have been well accomplished with the aid of spectroscopic, microscopic and XRD studies. The electrical performance studies of the OFET device, which was fabricated using the synthesized ZnO (as an active receptor layer) and P3HT (as channel layer) clearly revealed the selective sensing ability of CO gas with the sensitivity level reaching up to 25 ppm, which is, in fact, much below the prescribed OSHA standards. The sensitivity of the device is 6% (derived from the drain current of transfer characteristics) obtained at *V*_GS_ = −25 and *V*_DS_ = −40 V and the corresponding drain current sensitivity is 106 nA ppm^−1^. The novelty of the fabricated device originates from the fact that it functions in-open air and room temperature with excellent reversibility (reusability). Notably, the humidity seems to have little/no effect over the CO gas sensing abilities of the device. Therefore, this newly fabricated ZnO field-effect transistor, capable of detecting CO gas with the favourable sensitivity/slightly enhanced response characteristics, can be used in real-time air-quality examination and medical diagnosis perhaps in the form of electronic (nano)nose.

## Conflicts of interest

There is no conflict of interest.

## Supplementary Material
